# A Specific Tubular ApoA-I Distribution Is Associated to FSGS Recurrence after Kidney Transplantation

**DOI:** 10.3390/jcm10102174

**Published:** 2021-05-18

**Authors:** Conxita Jacobs-Cachá, Natàlia Puig-Gay, Ander Vergara, Maria-Alejandra Gabaldon, Joana Sellarés, Yolanda Villena-Ortiz, Irene Agraz, Francesc Moreso, Maria José Soler, Daniel Serón, Joan López-Hellín

**Affiliations:** 1 Nephrology Research Group, Nephrology Department, Vall d’Hebrón Research Institute (VHIR), Vall d’Hebron Barcelona Hospital Campus, Vall d’Hebrón Hospital Universitari, 08035 Barcelona, Spain; avergara@vhebron.net (A.V.); jsellares@vhebron.net (J.S.); iagraz@vhebron.net (I.A.); fjmoreso@vhebron.net (F.M.); m.soler@vhebron.net (M.J.S.); dseron@vhebron.net (D.S.); 2 Clinical Biochemistry Research Group, Department of Clinical Biochemistry, Vall d’Hebron Institut de Recerca (VHIR), Vall d’Hebron Barcelona Hospital Campus, Vall d’Hebron Hospital Universitari, 08035 Barcelona, Spain; n.puig.gay@gmail.com (N.P.-G.); yvillena@vhebron.net (Y.V.-O.); 3 Department of Pathology, Vall d’Hebron Barcelona Hospital Campus, Vall d’Hebron Hospital Universitari, 08035 Barcelona, Spain; magabaldon@vhebron.net; 4 Department of Medicine, Autonomous University of Barcelona, 08193 Barcelona, Spain

**Keywords:** focal segmental glomerulosclerosis, FSGS, recurrence, apolipoprotein A-I, apolipoprotein A-Ib, ApoA-I, ApoA-Ib, biomarkers, glomerular disease, kidney transplantation

## Abstract

A major complication of primary focal segmental glomerulosclerosis (FSGS) is its recurrence after kidney transplantation that happens in 30 to 40% of the patients. The diagnosis of these relapses is not always easy as the histological lesions are not highly specific and appear after the proteinuria increase. Currently, there are no accurate biomarkers to detect FSGS recurrence. Our group identified a modified form of Apolipoprotein A-I (ApoA-I), named ApoA-Ib, specifically present in the urine of recurrent FSGS patients after kidney transplantation. Aberrant forms of ApoA-I have also been described in the urine of native primary FSGS patients; this feature has been associated with prominent staining of ApoA-I at the apical membrane of the tubular cells. In this study, we aim to analyze the ApoA-I distribution in kidney allograft biopsies of recurrent FSGS patients. We detected ApoA-I by immunohistochemistry in kidney allograft biopsies of patients with FSGS relapse after kidney transplantation and in kidney allograft biopsies of patients with a disease different from FSGS in the native kidney (non-FSGS). In recurrent FSGS patients, ApoA-I was prominently localized at the brush border of the tubular cells, while in the non-FSGS patients, ApoA-I was found along the cytoplasm of the tubular cells. The localization of ApoA-I at the brush border of the tubular cells is a specific feature of primary FSGS in relapse. This suggests that ApoA-I staining in kidney biopsies, coupled with ApoA-Ib measurement in urine, could be used as a diagnostic tool of primary FSGS relapse after kidney transplantation due to its highly specific tubular distribution.

## 1. Introduction

The recurrence of primary focal segmental glomerulosclerosis (FSGS) is a major complication after kidney transplantation that happens in 30 to 40% of patients. FSGS relapse is noted by a sudden increase of proteinuria and confirmed by the detection of the characteristic histological pattern (partial scarring of some glomeruli) in a kidney allograft biopsy [1,2,3]. The diagnostic process of this entity is not always easy as the lesions usually appear weeks after the increase of the proteinuria and sampling errors can happen during the biopsy procedure and processing due to the focal nature of the disease. For these reasons, specific FSGS biomarkers are required to increase the reliability of the early diagnosis of FSGS relapses. In a previous study, we described a high molecular weight form of apolipoprotein A-I (ApoA-I) specifically present in the urine of FSGS recurrent patients, that we named ApoA-Ib [4]. ApoA-Ib was independent of proteinuria levels as it appeared in urine of FSGS relapsing patients and not in FSGS-unrelated patients with similar levels of proteinuria. Results obtained in two independent cohorts have confirmed that the presence of ApoA-Ib in urine allows discriminating FSGS recurrent patients from patients with proteinuria caused by a different etiology with a sensitivity > 90% and a specificity > 87% [4,5]. Moreover, ApoA-Ib was detected before most of the studied FSGS recurrence episodes, even before transplantation, suggesting that it has a potential prognostic value to detect patients at risk of relapse [5].

In a recent report [6], Clark et al. have analyzed ApoA-I levels and ApoA-I isoforms in the urine of a pediatric cohort composed of 228 patients with various kidney disorders and 40 healthy individuals. Urinary ApoA-I levels were higher in the patients’ group when compared to the healthy individuals’ group. This increased levels of ApoA-I in urine were attributed to the inclusion of patients with proximal tubulopathies (4.8%), renal dysplasia/CAKUT (8%), glomerulonephritis (8%), and patients in relapse of minimal change disease (MCD) (4.8%) and FSGS (1.3%). The remaining patients (73.1 %) had similar urinary ApoA-I levels than the healthy individuals, including those MCD and FSGS patients in remission. Interestingly, high molecular weight (HMW) forms of ApoA-I were analyzed in the patients with elevated urinary ApoA-I, and these forms were mostly present in MCD and FSGS relapsing patients but far more abundant in FSGS relapsing patients. The origin of these HMW forms of ApoA-I is unclear, but the authors suggest that they result from urinary cross-linking reactions [6,7]. Moreover, ApoA-I was found increased in proximal tubules of both MCD and FSGS relapsing patients, but in FSGS relapsing patients, ApoA-I was predominantly located in the brush border of the tubular cells and colocalized with the cubilin/megalin transporter [6].

Aberrant urinary ApoA-I forms seem to be associated with primary FSGS relapse both in the native and in transplanted kidneys [4,5,6,8]. Moreover, preliminary data has shown that the specific localization of this protein in the brush border of the tubular cells seems to be a specific feature of primary FSGS [6]. It is possible that ApoA-I staining in kidney biopsies could be a tool to help in primary FSGS diagnosis. Therefore, in the present study, we aim to analyze the distribution of ApoA-I in kidney allograft biopsies of FSGS recurrent patients and determine whether it has the potential to be used as a diagnostic biomarker of FSGS relapse.

## 2. Experimental Section

### 2.1. Patients and Samples

We studied the presence of ApoA-Ib in urine and ApoA-I distribution in the diagnostic kidney allograft biopsy of four FSGS recurrent patients and in four kidney allograft biopsies of non-FSGS patients indicated for graft dysfunction (2), delayed graft function (1), or surveillance (1). From each patient, 15 mL of first void urine was collected and, after centrifugation at 1500× *g*, to eliminate the urinary sediment, it was stored at −80 °C until use. The kidney allograft biopsies were performed under ultrasound guidance using spring-loaded 16- and 18-G needles. The kidney sections used to analyze ApoA-I were obtained from the pathology department of our hospital in three-micrometer sections embedded in paraffin once performed the diagnostic procedures.

The protocol for sample collection, storage, and analysis was approved by the Vall d’Hebron Hospital Ethics Committee (PR-IR 103/2008) and informed consent was obtained from all participants. The study was conducted in accordance with the principles of the Declaration of Helsinki. The clinical and research activities being reported are consistent with the Principles of the Declaration of Istanbul as outlined in the “Declaration of Istanbul on Organ Trafficking and Transplant Tourism”.

### 2.2. Urinary ApoA-Ib Detection

ApoA-Ib was detected in the urine of the included patients as described previously [4,5]. Briefly, all urinary samples were concentrated using a 3 kDa cut-off centrifuge filter and protein was quantified using Bradford protein assay. Sixty micrograms were separated in each case in SDS-PAGE gels with tris-glycine-SDS buffer, transferred to PVDF membranes, and probed with anti-ApoA-I (rabbit polyclonal PAB8546, Abnova, Jhongli, Taiwan), plus HRP-antirabbit secondary antibody P0448, (Dako, Glostrup, Denmark). Proteins were detected by chemiluminescence (Luminata Forte, Millipore and detector LAS3000, Fujifilm, Tokyo, Japan).

### 2.3. Histological Analysis

Biopsies were paraffin-embedded and processed for routine light microscopy and stained with hematoxylin–eosin, periodic acid Schiff (PAS) and Masson’s trichrome. Sample adequacy and histological lesions were evaluated according to the last update of the Banff criteria by local pathologists. Samples for TEM were fixed in a 2.5% glutaraldehide in 0.2 M sodium cacodylate buffer (pH = 7.4) followed by 1% osmium tetraoxide. After dehydration, the samples were embedded in EPON resin. Kidney sections (80 nm thick) were contrasted with aqueous uranyl acetate solution and lead citrate. The extension of the podocyte foot process effacement was assessed by local pathologists.

### 2.4. Immunodetection of ApoA-I in Renal Biopsies

For immunohistochemistry, the kidney biopsy sections were stained for ApoA-I using the Benchmark XT automated system and the associated specific reagents (Ventana Medical Systems. Inc., Oro Valley, AZ, USA) following the manufacturer instructions. We used a rabbit monoclonal antibody against ApoA-I (ab52945, Abcam plc, Cambridge, UK) as the primary antibody at a dilution of 1:200 in DAKO REAL Antibody Diluent (ref. S2022, Dako, Glostrup, Denmark). Diaminobenzidine (DAB) was used as a chromogen and hematoxylin as a counterstain. Representative pictures were taken at 40× magnification. For immunofluorescence, after deparaffinization, epitope retrieval was performed by heating the sections in 10 mM citrate buffer (pH 6.0). The sections were then blocked with 5% bovine serum albumin for 1 h at room temperature and incubated with antibodies against ApoA-I (dilution 1:1000 of rabbit polyclonal PAB8546, Abnova, Jhongli, Taiwan) and megalin (dilution 1:50 of mouse monoclonal MAB9578, R&D Systems, Minneapolis, MN, USA) overnight at 4 °C. Afterwards, the sections were rinsed twice in PBS1x-0.1% tween 20 and incubated for 1 h at room temperature with alexa fluor conjugated secondary antibodies diluted 1:500 (568 goat anti-rabbit IgG (H + L) and 488 goat anti-rabbit IgG (H + L), Invitrogen, Carlsbad, CA, USA). Hoechst 33342 (Invitrogen, Carlsbad, CA, USA) was used to stain the nuclei. Images were taken at 63× magnification using a super-high-resolution confocal microscope LSM980 (Zeiss, Jena, Germany).

## 3. Results

### 3.1. Demographic and Baseline Characteristics

The demographic and clinical characteristics, primary diagnoses, histological diagnoses, and urinary ApoA-Ib presence/absence of the included patients are shown in Table 1. Four kidney transplanted patients with primary FSGS in the native kidney (patients one to four in Table 1) were biopsied after kidney transplantation due to significant proteinuria. All the FSGS recurrent patients analyzed, except patient 1, showed FSGS recurrence during the first year after kidney transplantation. FSGS recurrence was diagnosed by FSGS findings under light microscopy and/or diffused foot process effacement (>80% of the analyzed surface) on electron microscopy. FSGS scarring was observed in patients 1 and 4, while patients 2 and 3 had no signs of FSGS lesions when analyzed under light microscopy. Generalized foot process effacement was seen in all the FSGS recurrent patients using electronic microscopy except for patient number 1, which could not be studied due to an insufficient sample (Table 1). Moreover, all FSGS recurrent patients showed the presence of the ApoA-Ib in urine (Table 1), a specific biomarker for FSGS relapse. We also included four kidney transplanted patients with an underlying disease in the native kidney different from FSGS and with an allograft biopsy (patients 5 to 8 in Table 1). Biopsy was indicated due to allograft dysfunction, delayed graft function, or surveillance. All the non-FSGS patients showed some degree of humoral or cellular rejection with or without interstitial fibrosis and tubular atrophy (IFTA) but without FSGS in the kidney allograft biopsy. Urinary ApoA-Ib was also analyzed in these patients and was in all cases negative (Table 1).

### 3.2. ApoA-I Is Located on the Brush Border of the Tubular Cells of FSGS Recurrent Patients

**Figure 1:**
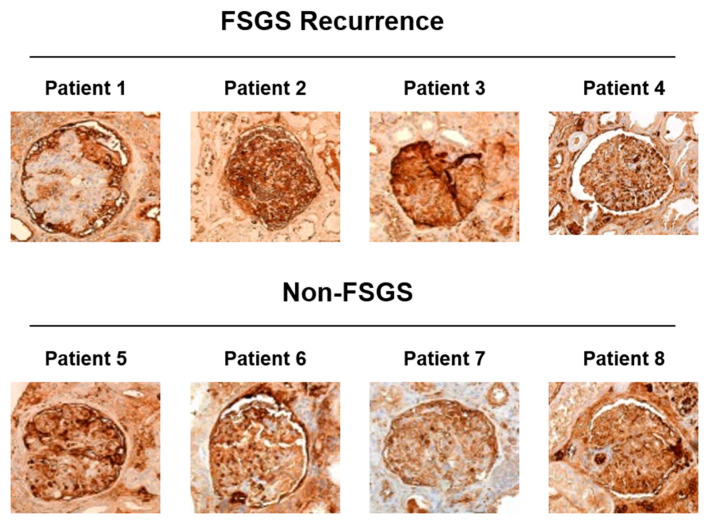
Glomerular ApoA-I staining in recurrent FSGS patients and non-FSGS patients. ApoA-I was detected by immunohistochemistry in kidney allograft biopsies of recurrent FSGS patients and non-FSGS patients. Images were taken at 40× magnification and a representative picture of the ApoA-I staining obtained in the glomerular compartment of each patient is shown. No differences in the glomerular ApoA-I staining were found between recurrent-FSGS and non-FSGS patients.

**Figure 2:**
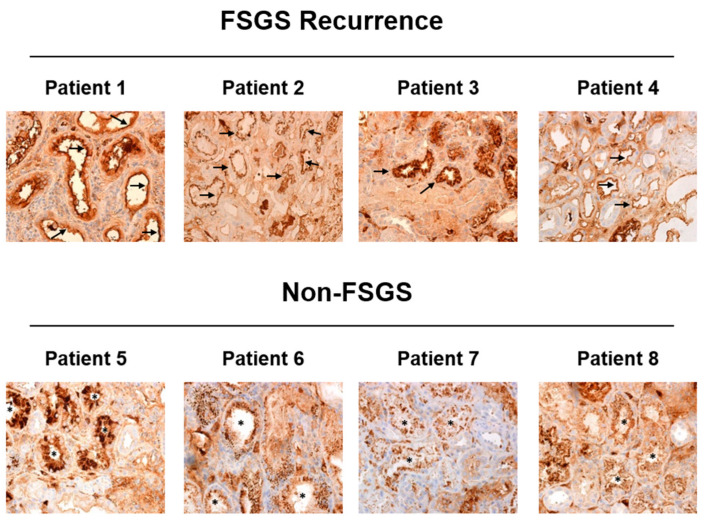
ApoA-I is located at the brush border of the tubular cells in FSGS recurrent patients. ApoA-I was detected by immunohistochemistry in kidney allograft biopsies of recurrent FSGS patients and non-FSGS patients. Images were taken at 40× magnification and a representative picture of the ApoA-I staining obtained in the tubular section of each patient is shown. In the tubular cells, ApoA-I was specifically found in the apical membrane of the tubular cells (brush border) in recurrent FSGS patients (arrows), while non-FSGS patients showed a vesicular ApoA-I staining pattern (asterisk).

**Figure 3:**
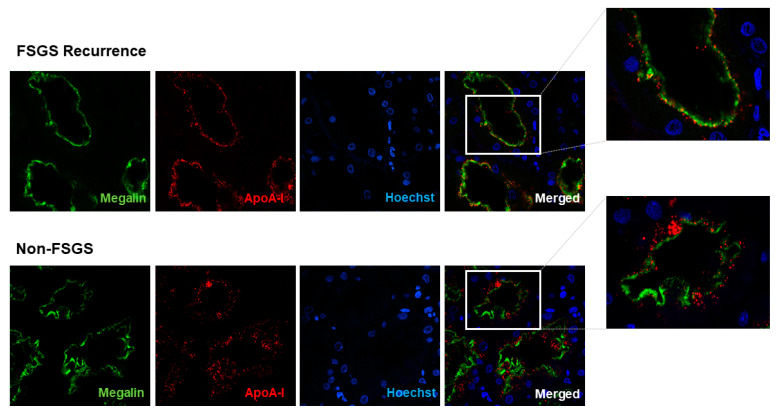
Localization of ApoA-I and megalin, a specific marker of the proximal tubular brush border, in recurrent FSGS and non-FSGS kidney allograft biopsies. ApoA-I and megalin were detected in kidney allograft biopsies by immunofluorescence using specific antibodies and nuclei were counterstained with Hoechst 33342. The figure shows a representative image of the megalin (in green), ApoA-I (in red) and Hoechst (in blue) staining obtained in recurrent-FSGS and non-FSGS patients; as well as the merged image. Megalin staining (in green) localized at the brush border of the proximal tubular cells in both recurrent FSGS and non-FSGS patients. Regarding ApoA-I, relapsing FSGS patients showed a strong ApoA-I-megalin colocalization, which was not observed in non-FSGS patients (see details of each case in the merged image zoom box).

ApoA-I was detected in the kidney allograft of the patients detailed in Table 1 by immunohistochemistry. We evaluated ApoA-I distribution in both the glomerular and the tubular compartment. As shown in Figure 1, all of the patients showed ApoA-I staining along the glomerular capillary tuft without differences between FSGS recurrence cases and non-FSGS cases. Regarding the tubular compartment, ApoA-I was found in most of the tubules both in FSGS recurrent patients and in non-FSGS patients but with a differential distribution between the two groups of patients (Figure 2). In the FSGS recurrent patients, ApoA-I was located at the brush border of the tubular cells and with low or no presence of ApoA-I in the cytoplasm (Figure 2, patients 1, 2, 3, and 4). Contrarily, the patients with a histological lesion different from FSGS, ApoA-I was found distributed from the apical membrane to the cytoplasm of the tubular cells, in most of the cases in a vesicular pattern (Figure 2, patients 5, 6, 7, and 8). To confirm these findings, we performed immunofluorescence detection of ApoA-I together with megalin, a protein specifically located at the brush border of the proximal tubular cells. As expected, in FSGS recurrent patients, ApoA-I and megalin prominently colocalized at the brush border of the proximal tubular cells (Figure 3). This was not observed in the non-FSGS patients where ApoA-I was mainly found in the cytoplasm of the tubular cells (Figure 3).

## 4. Discussion

In the present study, we have assessed the ApoA-I distribution in kidney allograft biopsies of four adult recurrent FSGS patients and four patients with an underlying cause of ESRD different from FSGS in the native kidney. ApoA-I was detected in both the glomerular and the tubular compartment. We found that the tubular ApoA-I distribution was clearly different between recurrent FSGS patients and non-FSGS patients (Figure 2 and Figure 3). In the non-FSGS kidney transplanted patients, ApoA-I was found in the tubular cells in a homogenous vesicular pattern suggestive of active reabsorption of ApoA-I. Contrarily, in the relapsing FSGS patients, ApoA-I was detected prominently at the brush border of the proximal tubular cells. However, no differences in the glomerular staining of ApoA-I were found (Figure 1). Our results are in concordance with the report of Clark et al. [6], supporting that ApoA-I reabsorption is impaired in primary FSGS patients. The previous study has also described that ApoA-I is located in the apical membrane of the tubular cells in kidney biopsies of 2 pediatric primary FSGS patients in relapse, a feature that seems to be characteristic of these patients [6].

One hypothesis to explain this finding is that the tubular function of the primary FSGS patients is impaired. Since the 1960s, it is well known that tubular and glomerular functions are interdependent [9,10], hence, tubular damage can sometimes be observed in patients with glomerular diseases [11,12]. Results obtained in in vitro models have demonstrated that proteinuria induces endoplasmic reticulum stress, an inflammatory state, and apoptosis upon the tubular cells [12,13,14,15]. It is largely known that the degree of persistent proteinuria is a predictor of loss of renal function in FSGS patients [16] and that proteinuria reduction improves renal outcomes [17]. In our study, all the included FSGS patients were biopsied due to FSGS relapse suspicion short after increased proteinuria was noted; hence the proteinuria evolution was short. Therefore, it is difficult that tubule-interstitial damage secondary to persistent proteinuria is happening in our relapsing FSGS patients. Moreover, the same degree of interstitial-tubular damage (IFTA I-II) was found in both recurrent FSGS and non-FSGS patients (Table 1), while the tubular ApoA-I staining was clearly different (Figure 2 and Figure 3). This fact suggests that tubular damage (when present) is hardly responsible for the retention of ApoA-I in the brush border of the tubular cells in recurrent FSGS patients (Figure 2 and Figure 3). Further, a recent report of Bu et al. [18] described that FSGS patients have increased staining of immunoglobulin G/albumin in tubular protein reabsorption droplets when compared to minimal change disease patients. This proves that, even in conditions of heavy proteinuria (>3.5 g/day), the tubular cells of the FSGS patients are actively reabsorbing proteins; hence it is improbable that ApoA-I is retained in the brush border of the tubular cells due to a failure in the reabsorption mechanisms.

Another possibility is that modifications that ApoA-I undergoes in primary FSGS, a feature described by our group [4,5] and other authors [6], impair the correct ApoA-I reabsorption in the proximal tubular cells. ApoA-I is found in blood, where it is associated with high-density lipoproteins (HDL) and is normally absent in the urine of healthy subjects due to tubular cubilin-mediated reabsorption [19,20]. In proteinuric kidney disease patients, ApoA-I is sometimes found in urine, probably due to failure of the tubular cells to reabsorb the totality of the low molecular weight filtered proteins [6]. A peculiarity of primary FSGS patients is that abnormal forms of ApoA-I are found in urine [4,5,6]. In a previous work, we detected a slightly higher molecular weight form of ApoA-I (ApoA-Ib) in the urine of FSGS recurrent patients. ApoA-Ib is far more abundant than other ApoA-I forms in the urine of FSGS recurrent patients [4,5]. The molecular characterization of ApoA-Ib has revealed a specific N-terminal end that is probably produced by a specific protease activity in primary FSGS patients [21]. Probably this specific N-terminal end of ApoA-Ib impairs the correct reabsorption of this ApoA-I form by an unknown mechanism. In line with this hypothesis, Clark et al. also suggest in their recent report that urinary HMW forms of ApoA-I produced by protein cross-linking would impair the correct reabsorption of ApoA-I in primary FSGS patients [6]. As per molecular weight, the urinary HMW forms of ApoA-I described in Clarks’ study are clearly not ApoA-Ib. But, attending to the urinary ApoA-I western blot images in their work, the main form of ApoA-I they find in the urine of primary FSGS patients is around 25 kDa [6], a molecular weight compatible with that of the ApoA-Ib form we also find in the urine of FSGS patients [21].

With the current data, it is difficult to underline the exact nature of the molecular mechanism that impairs ApoA-I reabsorption in primary FSGS patients, but it is a fact that ApoA-I can be detected in the tubular cells in a very specific pattern (Figure 2 and Figure 3). Our data, together with the data presented in Clark’s study [6], clearly points to the possibility of using ApoA-I staining in the diagnosis process of primary FSGS, both in the native kidney [6] and in recurrence after kidney transplantation. This discovery can be relevant for the current clinical practice since primary FSGS diagnosis is not straightforward and, further, there are no verified plasmatic biomarkers associated with primary FSGS [22]. Urinary ApoA-Ib, a biomarker described by us, is highly specific to detect FSGS relapses [4,5], but its use is limited as it cannot be measured in anuric patients. Therefore, other specific markers such as ApoA-I staining in kidney biopsies would be extremely useful, although it should be studied in a larger cohort of FSGS relapsing patients. We hope that our results encourage the scientific community to verify whether ApoA-I staining in kidney biopsies could be useful in primary FSGS diagnosis algorithm.

## Figures and Tables

**Table 1:** Demographic and clinical characteristics of the included patients. * Insufficient sample.

											Histology		
	Patient	Gender	Age (Years)	Native Kidney Disease	Transplant Number	Biopsy Indication	Time after KT (Years)	Proteinuria (g/24 h)	Serum Albumin (g/dL)	Serum Creatinine (mg/dL)	Light Microscopy	Electronic Microscopy	Urinary ApoA-Ib
**FSGS recurrence**	1	male	48	Primary FSGS	First	Proteinuria	1.67	4.1	4.26	2.18	FSGS	- *	Positive
	2	male	53	Primary FSGS	First	Proteinuria	0.75	9.6	3.32	1.86	IFTA II	>80% foot process effacement	Positive
	3	female	32	Primary FSGS	First	Proteinuria	0.60	1.2	3.4	1.62	IFTA II/Borderline rejection	>80% foot process effacement	Positive
	4	female	75	Primary FSGS	First	Proteinuria	0.06 (21 days)	2	3.92	2.46	FSGS/IFTA I	>80% foot process effacement	Positive
**Non-FSGS**	5	male	27	MPGN	First	Graft dysfunction	0.21	0.5	4.6	1.46	Acute cellular rejection IA/IFTA I	-	Negative
	6	male	49	Unknown cause of ESRD	Second	Delayed graft function	0.03 (10 days)	1	4.2	2.42	Borderline rejection	-	Negative
	7	male	45	Unknown cause of ESRD	First	Graft dysfunction /proteinuria	2.99	1.8	3.9	2.01	Chronic humoral rejection/Borderline rejection/ Arteriolar hyalinosis/IFTA II	-	Negative
	8	male	39	ESRD secondary to congenital kidney hypoplasia	Fourth	Surveillance	0.36	0.9	4.4	1.25	Acute humoral rejection	-	Negative

## Data Availability

The data presented in this study are available on request from the corresponding author.
